# Examining the Intersection of Ethnoracial Disparities and HIV Status in Substance Use Risks among U.S. Adults

**DOI:** 10.1007/s10461-024-04277-3

**Published:** 2024-01-31

**Authors:** Audrey Hang Hai, David Scott Batey, Christina S. Lee, Stacey Li, Rebecca Schnall

**Affiliations:** 1 School of Social Work, Tulane University, New Orleans, LA USA; 2 School of Social Work, Boston University, Boston, MA USA; 3 School of Public Health and Tropical Medicine, Tulane University, New Orleans, LA USA; 4 School of Nursing, Columbia University, New York, NY USA

**Keywords:** Racial disparities, Ethnic disparities, HIV, Substance use

## Abstract

Black/African American and Hispanic Americans experience significant HIV-related disparities. Substance use might be a contributing factor to these disparities, but there is limited research on this topic. This study investigated various substance use risks by HIV status and race/ethnicity (Black, Hispanic, White) among U.S. adults. We used data from the 2005–2019 National Survey on Drug Use and Health (*N* = 541,921). In each racial/ethnic group, the prevalence rates of past-year and past-month tobacco, alcohol, cannabis, and cocaine use, and past-year alcohol and illicit drug use disorders were estimated by HIV status. A series of logistic regressions with the interaction term of HIV x race/ethnicity were performed to examine race/ethnicity’s moderating effect on the HIV-substance use associations, while controlling for sociodemographic factors and survey year. Moderation analysis showed that HIV status’s association with the risks of past-year tobacco use (AOR = 1.67, 95% CI = 1.01–2.75), past-year cocaine use (AOR = 3.80, 95% CI = 1.91–7.57), past-month cocaine use (AOR = 5.34, 95% CI = 2.10–13.60), and past-year alcohol use disorder (AOR = 2.52, 95% CI = 1.29–4.92) differed significantly between Black and White adults. Between the Hispanic and White groups, HIV status’s association with the risks of past-year alcohol use (AOR = 2.00, 95% CI = 1.09–3.69), past-year cocaine use (AOR = 2.40, 95% CI = 1.06–5.39), and past-month cocaine use (AOR = 3.69, 95% CI = 1.36–10.02) also differed significantly. It is well-established that individuals with HIV face an elevated risk of substance use. Our study added valuable insights by highlighting that this phenomenon is particularly more significant among Black and Hispanic adults for several substances when compared to White adults. Implications for practice are discussed.

## Introduction

HIV is a significant public health issue affecting more than 1.2 million people in the United States (US) [1]. While the rate of new HIV diagnoses appears to be stabilizing [2], racial/ethnic disparities persist. In 2019, Black Americans accounted for 42.1% of all new HIV diagnoses despite comprising only 13% of the U.S. population [3]. Within all population groups, Black men who have sex with men (MSM) exhibit the highest rates of newly diagnosed HIV infections, AIDS diagnoses, and HIV/AIDS-related mortality rates [4]. Similarly, Hispanic Americans represented nearly 30% of all new HIV diagnoses in the United States [5]. When compared with the general population, Hispanics Americans are less aware of their HIV-positive status [6], have lower rates of utilizing preexposure prophylaxis (PrEP) [7], and receive HIV care at a significantly lower rate [8].

Substance use is widely recognized to be closely linked to HIV, with negative impacts on all aspects of the disease including transmission, diagnosis, clinical progression, and treatment [9]. Specifically, substance use acts as a vector for HIV transmission by increasing high-risk behaviors, such as the sharing of contaminated needles, participating in sex work, and engaging in unprotected sex [10, 11]. Among HIV positive individuals with substance use disorders (SUD), the effectiveness of universal HIV test-and-treat strategies is compromised [12, 13] and post-diagnosis linkage to care is less consistent [14]. Even when linked to care, HIV positive individuals with SUD exhibit inconsistent clinic attendance, delayed initiation of antiretroviral medication, poor adherence [15–18], and lower care retention [19–22]. Furthermore, substance use also directly impacts the progression of HIV disease by elevating viral load and inducing immunomodulation [10, 11].

Considering substance use’s significant negative impact across the continuum of HIV care, it is crucial to investigate its potential role in HIV-related disparities in Black and Hispanic populations. Currently, there are limited data on the co-occurrence of these syndemic health conditions. A review on past studies showed that estimates vary widely, with 21–74% of people with HIV (PWH) believed to have comorbid SUD [17, 23–27]. Even less is known about substance use among Black and Hispanic PWH and how it compares to non-HIV-infected individuals from the same racial/ethnic groups or their White counterparts. Conducting research in this area holds significant implications. If particular types of substance use and SUD are found to be more prevalent among PWH compared to their HIV-negative counterparts in the Black and Hispanic communities, it is reasonable to infer that these substance use issues may play a role in the HIV-related disparities experienced by these groups. As a result, identifying specific substance use challenges provides a clearer direction for targeted interventions aimed at mitigating HIV-related racial/ethnic disparities.

As a step to address this research gap, the present study utilized nationally representative data to compare the prevalence of substance use and SUD across different racial/ethnic groups (Black, Hispanic, and White Americans) and HIV status among U.S. adults. Examined substances included tobacco use, alcohol use, cannabis use, cocaine use, alcohol use disorder, and illicit drug use disorder. It is important to note that while a comprehensive list of substances would be ideal, our selection was based on previous research on the most common types of substance use [27] and the availability of data [28].

## Methods

### Data and Sample

This study analyzed data gathered from the National Survey on Drug Use and Health (NSDUH). The NSDUH is a comprehensive nationwide survey conducted annually in all 50 states of the US and the District of Columbia. This survey employs a multi-stage probability sampling design to gather nationally representative data from non-institutionalized civilians aged 12 years and older. The primary objective is to collect comprehensive data on various health topics including substance use and mental health. More detailed information about the survey design and methodology can be obtained elsewhere [28]. For this present study, we analyzed data collected between 2005 and 2019, consisting of 541,921 adults aged 18 and above. This study was a secondary analysis using publicly available anonymized data and was thereby exempt from Institutional Review Board or other ethical compliance requirements.

### Measures

#### Substance use

We examined self-reported past-year and past-month tobacco, alcohol, cannabis, and cocaine use, and past-year alcohol and illicit drug use disorders based on the Diagnostic and Statistical Manual of Mental Disorders (DSM-IV) (yes, no) [29].

#### HIV status

Respondents were asked if they had ever been told that they had HIV or AIDS (yes, no).

#### Race/ethnicity

Self-identified races/ethnicities included Non-Hispanic (NH) White, NH Black/African American, and Hispanic.

#### Sociodemographic variables

included age in years (18–29, 30–49, 50 or older), gender (female, male), family income (living in poverty, 1-2x the federal poverty threshold, >2x the federal poverty threshold), education (less than high school, high school graduate, some college/associate degree, college graduate), marital status (married, divorced/separated/widowed, never been married), and urbanicity (large metro, small metro, non-metro [classified based on the Metropolitan Area standards] [30]).

### Data Analysis

All statistical analyses were weighted to account for the NSDUH’s stratified cluster sampling design and carried out using Stata 18SE [31]. First, we calculated descriptive statistics to characterize the sample by HIV status. Second, we estimated the prevalence rates of substance use and SUDs in the HIV and no-HIV samples separately. We also conducted a series of logistic regressions to assess the association of HIV status with substance use risks, while adjusting for sociodemographic controls and survey year. Third, logistic regressions with the interaction term of HIV x race/ethnicity were performed to examine race/ethnicity’s moderating effect on the HIV-substance use associations, while controlling for sociodemographic factors and survey year.

## Results

### Sample Characteristics

Table 1 presents the demographic characteristics of the sample by HIV status. US adults without HIV were largely comprised of NH White individuals, with approximately 72% who self-identified as NH White (71.82%), 12% as NH Black (12.44%), and 16% as Hispanic (15.73%). More racial/ethnic minorities constituted the population with HIV, including about half self-identified as NH White (51.15%), 30% as NH Black (29.62%), and 19% as Hispanic (19.23%). Compared to the group without HIV, a higher percentage of the group with HIV were middle aged (30–49 years old) (HIV vs. No HIV: 45.65% vs. 34.72%) and a lower percentage were young adults (18–29 years old) (HIV vs. No HIV: 11.64% vs. 21.32%). About half of the US adults without HIV (48.14%) and 80% of those with HIV (80.17%) identified as male. The HIV and non-HIV group compositions in terms of education levels appeared relatively similar with slight differences on the less than high school (HIV vs. No HIV: 18.21% vs. 14.37%) and high school graduate levels (HIV vs. No HIV: 21.15% vs. 29.15%). More PWH lived in poverty (HIV vs. No HIV: 25.69% vs. 13.22%), had never been married (HIV vs. No HIV: 64.81% vs. 26.73%), and resided in large metropolitan areas (HIV vs. No HIV: 74.49% vs. 53.29%).

**Table 1:** Sociodemographic characteristics of U.S. adults by HIV status, 2005–2019 national survey on drug use and health (*N* = 541,921)

	% [95% CI]	
	No HIV (*N* = 534,777)	HIV (*N* = 921)
*Race/Ethnicity*		
NH White	71.82 [71.52, 72.12]	51.15 [45.83, 56.44]
NH Black/African American	12.44 [12.22, 12.67]	29.62 [25.01, 34.69]
Hispanic	15.73 [15.49, 15.97]	19.23 [15.38, 23.77]
*Age*		
18–29	21.32 [21.14, 21.51]	11.64 [9.34, 14.40]
30–49	34.72 [34.48, 34.97]	45.65 [41.20, 50.18]
50 or older	43.96 [43.64, 44.27]	42.71 [38.53, 46.99]
*Gender*		
Female	51.86 [51.66, 52.07]	19.83 [16.56, 23.56]
Male	48.14 [47.93, 48.34]	80.17 [76.44, 83.44]
*Family income*		
Living in poverty	13.22 [13.05, 13.39]	25.69 [21.19, 30.79]
1-2x federal poverty line	19.58 [19.40, 19.76]	24.71 [20.51, 29.46]
>2x federal poverty line	67.20 [66.93, 67.47]	49.59 [43.77, 55.43]
*Education*		
Less than high school	14.37 [14.18, 14.56]	18.21 [14.66, 22.39]
High school graduate	29.15 [28.90, 29.41]	21.15 [17.69, 25.09]
Some college/associate degree	27.93 [27.74, 28.13]	30.66 [26.87, 34.73]
College graduate	28.54 [28.24, 28.84]	29.98 [24.85, 35.66]
*Marital status*		
Married	53.07 [52.79, 53.34]	16.67 [13.28, 20.73]
Divorced/separated/widowed	20.20 [20.00, 20.41]	18.52 [15.15, 22.43]
Never been married	26.73 [26.52, 26.94]	64.81 [60.28, 69.09]
*Urbanicity*		
Large metro	53.29 [52.94, 53.65]	74.49 [69.99, 78.52]
Small metro	30.60 [30.23, 30.96]	16.79 [13.73, 20.38]
Non-metro	16.11 [15.87, 16.36]	8.72 [6.51, 11.57]

### Tobacco Use Risk

Table 2 presents past-year and past-month substance use prevalence by race/ethnicity and HIV status. Among NH White adults, the prevalence rate of past-year tobacco use was 48.06% (95% CI = 40.62–55.59) for PWH and 34.86% (95% CI = 34.59–35.13) for people without HIV (PWOH). Among NH Black adults, the prevalence of past-year tobacco use was 54.47% (95% CI = 46.60-62.12) for PWH and 32.65% (95% CI = 32.08–33.23) for PWOH. Among Hispanic adults, the prevalence of past-year tobacco use was 48.21% (95% CI = 37.08–59.52) for PWH and 26.84% (95% CI = 26.37–27.32) for PWOH. Moderation analysis showed that HIV status’s association with past-year tobacco use risks differed significantly between NH Black and NH White adults (NH Black x HIV AOR = 1.67, 95% CI = 1.01–2.75). While HIV status was not significantly associated with past-year tobacco use risks among NH White adults, PWH had a significantly higher risk for past-year tobacco use than PWOH among NH Black (AOR = 1.76, 95% CI = 1.24–2.49) and Hispanic adults (AOR = 1.78, 95% CI = 1.15–2.75).

**Table 2:** Substance use risk by HIV status and race/ethnicity among U.S. adults, 2005–2019 national survey on drug use and health (*N* = 541,921)

	% [95% CI]		HIV vs. No HIV		HIV x Race/Ethnicity	
	No HIV (*N* = 534,777)	HIV (*N* = 921)	AOR	95% CI	AOR	95% CI
*Past-year tobacco use*						
NH White	34.86 [34.59, 35.13]	48.06 [40.62, 55.59]	1.26	0.89-1.79	1.00	-
NH Black/African American	32.65 [32.08, 33.23]	54.47 [46.60, 62.12]	**1.76**	**1.24–2.49**	**1.67**	**1.01–2.75**
Hispanic	26.84 [26.37, 27.32]	48.21 [37.08, 59.52]	**1.78**	**1.15–2.75**	1.67	0.92-3.02
*Past-month tobacco use*						
NH White	29.51 [29.25, 29.76]	41.71 [34.55, 49.22]	1.29	0.91-1.82	1.00	-
NH Black/African American	28.67 [28.15, 29.19]	48.61 [40.46, 56.84]	**1.66**	**1.16–2.38**	1.51	0.91-2.51
Hispanic	21.17 [20.71, 21.63]	38.05 [27.90, 49.36]	**1.61**	**1.03–2.52**	1.51	0.81-2.82
*Past-year alcohol use*						
NH White	74.36 [74.11, 74.61]	75.93 [70.17, 80.88]	0.97	0.69-1.35	1.00	-
NH Black/African American	62.03 [61.38, 62.68]	65.34 [57.00, 72.83]	1.17	0.80-1.69	1.56	0.96-2.53
Hispanic	63.68 [63.15, 64.20]	79.73 [69.64, 87.09]	1.64	0.94-2.84	**2.00**	**1.09–3.69**
*Past-month alcohol use*						
NH White	60.45 [60.17, 60.73]	64.18 [57.94, 69.98]	0.96	0.72-1.28	1.00	-
NH Black/African American	46.93 [46.32, 47.53]	52.10 [44.27, 59.82]	1.19	0.84-1.68	1.52	0.96-2.42
Hispanic	47.28 [46.73, 47.84]	58.12 [45.59, 69.68]	1.07	0.64-1.81	1.23	0.71-2.11
*Past-year cannabis use*						
NH White	12.92 [12.76, 13.08]	36.74 [30.36, 43.62]	**2.70**	**1.94–3.75**	1.00	-
NH Black/African American	15.04 [14.68, 15.42]	34.56 [28.13, 41.61]	**2.59**	**1.89–3.55**	1.01	0.64-1.61
Hispanic	10.80 [10.53, 11.08]	28.69 [19.73, 39.71]	**2.66**	**1.48–4.79**	0.93	0.48-1.78
*Past-month cannabis use*						
NH White	7.96 [7.85, 8.08]	28.55 [22.85, 35.03]	**3.06**	**2.17–4.32**	1.00	-
NH Black/African American	9.88 [9.59, 10.18]	26.32 [20.65, 32.89]	**2.75**	**1.98–3.83**	0.90	0.54 − 1.50
Hispanic	6.53 [6.30, 6.76]	15.87 [9.57, 25.16]	**2.04**	**1.08–3.83**	0.63	0.31-1.27
*Past-year cocaine use*						
NH White	2.14 [2.08, 2.19]	8.41 [5.68, 12.29]	**2.40**	**1.55–3.73**	1.00	-
NH Black/African American	1.84 [1.69, 1.99]	16.36 [11.27, 23.15]	**6.02**	**3.75–9.66**	**3.80**	**1.91–7.57**
Hispanic	2.10 [1.99, 2.22]	15.87 [8.86, 26.81]	**6.77**	**3.31–13.83**	**2.40**	**1.06–5.39**
*Past-month cocaine use*						
NH White	0.72 [0.69, 0.75]	2.94 [1.46, 5.85]	1.96	0.95-4.06	1.00	-
NH Black/African American	0.95 [0.84, 1.06]	11.95 [7.63, 18.24]	**7.46**	**4.43–12.55**	**5.34**	**2.10–13.60**
Hispanic	0.77 [0.70, 0.85]	9.21 [4.40, 18.28]	**8.68**	**3.92–19.23**	**3.69**	**1.36–10.02**
*Past-year alcohol use disorder*						
NH White	7.08 [6.96, 7.20]	10.31 [6.97, 15.00]	0.95	0.60-1.49	1.00	-
NH Black/African American	6.15 [5.91, 6.40]	15.30 [9.98, 22.74]	**2.12**	**1.27–3.54**	**2.52**	**1.29–4.92**
Hispanic	7.12 [6.89, 7.36]	13.62 [7.79, 22.73]	1.36	0.72-2.54	1.42	0.62-3.25
*Past-year illicit drug use disorder*						
NH White	2.55 [2.48, 2.62]	11.11 [8.04, 15.16]	**3.27**	**2.30–4.65**	1.00	-
NH Black/African American	3.50 [3.31, 3.70]	13.19 [8.12, 20.73]	**3.19**	**1.76–5.76**	1.09	0.57-2.09
Hispanic	2.61 [2.46, 2.77]	16.23 [10.21, 24.81]	**5.78**	**3.25–10.28**	1.75	0.92-3.36

*Note* Data from years 2005–2019 are pooled. Adjusted Odds ratios (AOR) were estimated with year and sociodemographic factors adjusted for (age, gender, household income, education, marital status, and urbanicity). Bolded AOR are statistically significant at *p* < .050. All estimates adjusted for the NSDUH’s complex sampling design

Although the race/ethnicity x HIV status interaction did not reach statistical significance for past-month tobacco use, similar patterns were observed. HIV status was a significant predictor of past-month tobacco use for NH Black (AOR = 1.66, 95% CI = 1.16–2.38) and Hispanic adults (AOR = 1.61, 95% CI = 1.03–2.52) but not for NH White adults. Among NH White adults, the prevalence rate of past-month tobacco use was 41.71% (95% CI = 34.55–49.22) for PWH and 29.51% (95% CI = 29.25–29.76) for PWOH. Among NH Black adults, the prevalence of past-month tobacco use was 48.61% (95% CI = 40.46–56.84) for PWH and 28.67% (95% CI = 28.15–29.19) for PWOH. Among Hispanic adults, the prevalence of past-month tobacco use was 38.05% (95% CI = 27.90-49.36) for PWH and 21.17% (95% CI = 20.71–21.63) for PWOH.

### Alcohol Use Risk

Among NH White adults, 75.93% (95% CI = 70.17–80.88) of PWH and 74.36% (95% CI = 74.11–74.61) of PWOH reported past-year alcohol use; 64.18% (95% CI = 57.94–69.98) of PWH and 60.45% (95% CI = 60.17–60.73) of PWOH reported past-month alcohol use. Among NH Black adults, 65.34% (95% CI = 57.00-72.83) of PWH and 62.03% (95% CI = 61.38–62.68) of PWOH reported past-year alcohol use; 52.10% (95% CI = 44.27–59.82) of PWH and 46.93% (95% CI = 46.32–47.53) of PWOH reported past-month alcohol use. Among Hispanic adults, 79.73% (95% CI = 69.64–87.09) of PWH and 63.68% (95% CI = 63.15–64.20) of PWOH reported past-year alcohol use; 58.12% (95% CI = 45.59–69.68) of PWH and 47.28% (95% CI = 46.73–47.84) of PWOH reported past-month alcohol use. Moderation analysis suggested that HIV status’s association with past-year alcohol use risks differed significantly between the Hispanic and NH White groups (Hispanic x HIV AOR = 2.00, 95% CI = 1.09–3.69), although HIV status was not significantly associated with higher risk of past-year or past-month alcohol use in any of the three racial/ethnic groups.

### Cannabis Use Risk

Among NH White adults, 36.74% (95% CI = 30.36–43.62) of PWH and 12.92% (95% CI = 12.76–13.08) of PWOH reported past-year cannabis use; 28.55% (95% CI = 22.85–35.03) of PWH and 7.96% (95% CI = 7.85–8.08) of PWOH reported past-month cannabis use. Among NH Black adults, 34.56% (95% CI = 28.13–41.61) of PWH and 15.04% (95% CI = 14.68–15.42) of PWOH reported past-year cannabis use; 26.32% (95% CI = 20.65–32.89) of PWH and 9.88% (95% CI = 9.59–10.18) of PWOH reported past-month cannabis use. Among Hispanic adults, 28.69% (95% CI = 19.73–39.71) of PWH and 10.80% (95% CI = 10.53–11.08) of PWOH reported past-year cannabis use; 15.87% (95% CI = 9.57–25.16) of PWH and 6.53% (95% CI = 6.30–6.76) of PWOH reported past-month cannabis use. No significant racial/ethnic differences were found for HIV status’ relationship with past-year or past-month cannabis use risks. Compared to PWOH, PWH reported a significantly higher risk for past-year or past-month cannabis use in all three racial/ethnic groups (Past-year cannabis use: NH White AOR = 2.70, 95% CI = 1.94–3.75; NH Black AOR = 2.59, 95% CI = 1.89–3.55; Hispanic AOR = 2.66, 95% CI = 1.48–4.79) (Past-month cannabis use: NH White AOR = 3.06, 95% CI = 2.17–4.32; NH Black AOR = 2.75, 95% CI = 1.98–3.83; Hispanic AOR = 2.04, 95% CI = 1.08–3.83).

### Cocaine Use Risk

Among NH White adults, 8.41% (95% CI = 5.68–12.29) of PWH and 2.14% (95% CI = 2.08–2.19) of PWOH reported past-year cocaine use. Among NH Black adults, 16.36% (95% CI = 11.27–23.15) of PWH and 1.84% (95% CI = 1.69–1.99) of PWOH reported past-year cocaine use. Among Hispanic adults, 15.87% (95% CI = 8.86–26.81) of PWH and 2.10% (95% CI = 1.99–2.22) of PWOH reported past-year cocaine use. Past-year cocaine use risks were significantly higher for PWH than PWOH in all three racial/ethnic groups. However, this elevated risk is significantly more pronounced among NH Black and Hispanic adults than NH White adults (NH Black x HIV AOR = 3.80, 95% CI = 1.91–7.57; Hispanic x HIV AOR = 2.40, 95% CI = 1.06–5.39). Adjusting for demographic factors, the odds of past-year cocaine use for PWH is more than six times the odds for PWOH in NH Black (AOR = 6.02, 95% CI = 3.75–9.66) and Hispanic populations (AOR = 6.77, 95% CI = 3.31–13.83), whereas the AOR for NH White adults was less than three (AOR = 2.40, 95% CI = 1.55–3.73).

Among NH White adults, 2.94% (95% CI = 1.46–5.85) of PWH and 0.72% (95% CI = 0.69-0.75) of PWOH reported past-month cocaine use. Among NH Black adults, 11.95% (95% CI = 7.63–18.24) of PWH and 0.95% (95% CI = 0.84-1.06) of PWOH reported past-month cocaine use. Among Hispanic adults, 9.21% (95% CI = 4.40-18.28) of PWH and 0.77% (95% CI = 0.70-0.85) of PWOH reported past-month cocaine use. Significant racial/ethnic differences were also found for past-month cocaine use (NH Black x HIV AOR = 5.34, 95% CI = 2.10–13.60; Hispanic x HIV AOR = 3.69, 95% CI = 1.36–10.02). Living with HIV was significantly linked to elevated risks for past-month cocaine use among NH Black (AOR = 7.46, 95% CI = 4.43–12.55) and Hispanic adults (AOR = 8.68, 95% CI = 3.92–19.23). In contrast, no significant HIV-past-month-cocaine-use link was found among NH White adults.

### Alcohol Use Disorder Risk

Among NH White adults, the prevalence rate of past-year alcohol use disorder was 10.31% (95% CI = 6.97-15.00) for PWH and 7.08% (95% CI = 6.96–7.20) for PWOH. Among NH Black adults, the prevalence of alcohol use disorder was 15.30% (95% CI = 9.98–22.74) for PWH and 6.15% (95% CI = 5.91–6.40) for PWOH. Among Hispanic adults, the prevalence of alcohol use disorder was 13.62% (95% CI = 7.79–22.73) for PWH and 7.12% (95% CI = 6.89–7.36) for PWOH. Moderation analysis suggested that HIV status’s association with past-year alcohol use disorder differed significantly between the NH Black and NH White groups (NH Black x HIV AOR = 2.52, 95% CI = 1.29–4.92). NH Black adults with HIV reported significantly greater risks for past-year alcohol use disorder than NH Black adults without HIV (AOR = 2.12, 95% CI = 1.27–3.54), whereas there was no significant association between HIV status and past-year alcohol use disorder for Hispanic or NH White adults.

### Illicit Drug Use Disorder Risk

Among NH White adults, the prevalence rate of past-year illicit drug use disorder was 11.11% (95% CI = 8.04–15.16) for PWH and 2.55% (95% CI = 2.48–2.62) for PWOH. Among NH Black adults, the prevalence of illicit drug use disorder was 13.19% (95% CI = 8.12–20.73) for PWH and 3.50% (95% CI = 3.31–3.70) for PWOH. Among Hispanic adults, the prevalence of illicit drug use disorder was 16.23% (95% CI = 10.21–24.81) for PWH and 2.61% (95% CI = 2.46–2.77) for PWOH. PWH had a greater risk for past-year illicit drug use disorder than PWOH among NH White (AOR = 3.27, 95% CI = 2.30–4.65), NH Black (AOR = 3.19, 95% CI = 1.76–5.76), and Hispanic adults (AOR = 5.78, 95% CI = 3.25–10.28). There were no significant racial/ethnic differences.

## Discussion

The syndemic of HIV and substance use poses a more serious challenge among Black and Hispanic Americans [4, 6]. In addition to critical factors such as racism, economic marginalization, and limited access to high-quality HIV care [4], higher prevalence of certain substances among those living with HIV might be another contributing factor to the observed HIV-related disparities in the Black and Hispanic communities. This study investigated the risks of various substance use behaviors by HIV status and race/ethnicity among US adults. Our findings indicated that compared to NH White adults, the associations between HIV status and higher risks of tobacco use, cocaine use, and AUD are stronger among NH Black adults. Additionally, Hispanic adults exhibited stronger associations between HIV status and higher risks of alcohol use and cocaine use.

### Tobacco

This study found that Black adults with HIV are more likely to report tobacco use when compared to Black adults without HIV. In contrast, there was no significant difference in tobacco use risk based on HIV status for White adults. This finding is consistent with previous research indicating that low-income HIV positive Black Americans present to HIV treatment with high rates of health compromising behaviors such as tobacco use [32]. This also suggests that tobacco use might be a contributing factor to the HIV-related disparities faced by the Black community. Tobacco use has been found to be associated with a myriad of serious consequences among HIV-positive people, including higher viral loads, lower CD4 + cell counts [33, 34], increased incidence of opportunistic infections, cardiovascular and lung disease, human papilloma virus (HPV) associated cancers, and increased overall mortality [35–37]. In addition, nicotine dependence has emerged as a significant and independent barrier to adherence to antiretroviral therapy in HIV-positive smokers [18].

Limited research exists on the relationship between tobacco use and HIV outcomes specifically in the population of Black adults with HIV. However, existing research has shed light on the barriers and motivators to smoking cessation among HIV-positive Black MSMs who smoke. These barriers include low self-efficacy and environmental, cultural, emotional, and provider factors. On the other hand, motivators for smoking cessation include financial considerations, health concerns, appearance, and the changing social norms surrounding smoking [38]. Given the limited research, there is a pressing need for more studies to develop effective and scalable smoking cessation interventions tailored to the unique needs of Black adults with HIV. Such interventions hold the potential to reducing the HIV-related disparities experienced by the Black community.

### Alcohol

This study suggested that higher prevalence of alcohol problems might be a common contributing factor to HIV-related disparities for both Black and Hispanic populations. Our study revealed that Black adults showed stronger links between being HIV positive and increased risks of AUD compared to NH White adults. Similarly, Hispanic adults exhibit stronger associations between being HIV positive and elevated risks of alcohol use than NH White adults. Research on alcohol use among Black and Hispanic PWH is scant. However, our finding is generally in line with previous studies. For example, Lipira et al. found that among Black women living with HIV, alcohol use and misuse are common and correlated with poor HIV-related outcomes, such as lower likelihood of ART adherence and lack of viral suppression [39]. Further, not only do Black and Hispanic adults with HIV exhibit a higher prevalence of alcohol issues, but they might also experience a greater burden from these problems. Data from a nationally representative alcohol survey showed that Black and Hispanic drinkers are more likely than White drinkers to report social consequences of drinking, regardless of drinking levels and particularly at lower levels of drinking [40]. Further research is needed to better understand the intricacies of these associations and their implications and to develop targeted interventions that address alcohol-related disparities within Black and Hispanic communities with HIV.

### Cocaine

The present study indicated that cocaine use might be another factor that plays a role in the HIV-related disparities among both Black and Hispanic populations. Our findings show that the odds of cocaine use among HIV-positive White adults are roughly twice as high as those among White adults without HIV. In contrast, for Black and Hispanic adults, this disparity is significantly greater, with odds about six to eight times higher (HIV + vs. HIV- in the odds of cocaine use). This finding is consistent with the limited existing research on cocaine use among Black and Hispanic PWH. Notably, previous research indicates a link between cocaine use and worsened HIV outcomes for these populations. For instance, Sharpe et al. found that crack use was more frequently reported among Black HIV-infected women compared to those of other racial/ethnic groups [41]. Among Black women prescribed ART medicines, crack users and users of other drugs reported greater difficulty adhering to ART compared to non-users [41]. Additionally, cocaine use was associated with a decline in body mass index (BMI)—an independent risk factor for HIV-related mortality—among HIV-infected Hispanic men, but not among their uninfected counterparts [42]. More research in this area is critically needed.

### Limitations

The findings of this study should be considered within the context of its limitations. First, it is important to note that the NSDUH data, although collected annually, are cross-sectional in nature. As a result, drawing causal inferences based on this data was not possible. Second, our reliance on respondent self-report introduced potential biases. There is a possibility that the prevalence of substance use in certain populations was underestimated due to social desirability and recall biases. Recent research also suggests that errors in national surveys like NSDUH, including selective nonresponse and underreporting, contribute significantly to underestimations of stigmatized substance use behaviors [43]. Third, the scope of the NSDUH data did not permit examinations of sociocultural factors that are particularly pertinent to Black and Hispanic populations, such as racism and acculturation. In addition, the constraints of the available data prevented the inclusion of several critical confounders in our analyses, such as participant geographic location, which has been found to be a source of considerable variability in substance use prevalence rates [22].

## Conclusions

The syndemic of HIV and substance use has a disproportionate impact on Black and Hispanic Americans. It is well-established that individuals with HIV face an elevated risk of substance use. Our study added valuable insights by highlighting that this phenomenon is particularly more significant among Black and Hispanic adults for several substances when compared to White adults. Particularly, the use of tobacco, alcohol, and cocaine might play a role in the HIV-related disparities observed among Black Americans. Similarly, alcohol and cocaine use could be linked to the HIV-related disparities experienced by Hispanic Americans. Our findings carry important implications: integrated strategies that simultaneously tackle both HIV and the identified substance use issues could play a pivotal role in alleviating these racial/ethnic disparities. These integrated approaches might involve routine assessment of substance use within HIV clinical settings and the provision of integrated treatment targeting both substance use and HIV. Additionally, the development of culturally tailored, evidence-based integrated interventions could proactively address challenges related to antiretroviral therapy adherence and achieving viral suppression within Black and Hispanic communities.
